# Promoting the effect of microbubble-enhanced ultrasound on hyperthermia in rabbit liver

**DOI:** 10.1007/s10396-021-01187-y

**Published:** 2022-01-24

**Authors:** Yuwen Yang, Huanqian Luo, Yang Zhao, Lu Li, Yan He, Fen Xi, Hai Jin, Ruru Gao, Qiong Luo, Jianhua Liu

**Affiliations:** 1 The First Affiliated Hospital of Jinan University, Guangzhou, China; 2 Department of Medical Ultrasound, Guangzhou First People’s Hospital, School of Medicine, South China University of Technology, Guangzhou, China; 3 Department of Medical Ultrasound, Dongguan People’s Hospital, Southern Medical University, Dongguan, China; 4 Department of Ultrasound, Xinqiao Hospital, Chongqing, China

**Keywords:** Cavitation, Hyperthermia, Liver, Microbubble, Microbubble-enhanced ultrasound

## Abstract

**Purpose:**

The heat-sink effect is one reason for the insufficient temperature increase in hyperthermia (HT) treatment for cancer. Microbubbles (MBs) nucleate inertial cavitation under therapeutic ultrasound (TUS) exposure, which form microbubble-enhanced ultrasound (MEUS), which results in blocking blood perfusion in the targeted liver tissues. This study aimed to determine if synergistic effects exist during HT in the liver when combined with MEUS.

**Methods:**

Forty rabbits with surgically exposed livers were randomly divided into TUS + MB + HT, MB + HT, normal saline + HT, and MB + sham groups (*n* = 10 in each group). Liver perfusion was evaluated using contrast-enhanced ultrasound. The temperatures of the liver tissues were monitored using thermocouples. Pathological changes were determined by hematoxylin and eosin (H&E) staining. Serum hepatic transaminases were evaluated.

**Results:**

MEUS pretreatment almost completely blocked the perfusion of targeted areas. The TUS + MB + HT and MB + HT groups showed significantly higher temperatures in treated areas than those in the other groups. However, the TUS + MB + HT group exhibited a more stable and regular increase in temperatures in the fitting curves compared with the MB + HT group. H&E staining revealed swelling hepatocytes, hemorrhage, and thrombosis in the portal area in the TUS + MB + HT group.

**Conclusion:**

MEUS reduced the blood perfusion in the targeted liver tissues, and, therefore, overcame the heat-sink effect during the HT procedure in rabbits. MEUS pretreatment might have the potential to enhance the therapeutic effect of HT.

## Introduction

Hepatocellular carcinoma (HCC) is one of the most common malignancies worldwide and is a leading cause of cancer-related mortality [1]. Traditional curative strategies include surgical resection, liver transplantation, chemotherapy, radiation therapy, and thermal therapy [2, 3]. The appropriate selection of curative strategies contributes to the overall survival rates in patients. The optimal choice of curative surgery for patients with HCC mainly depends on the early stage tumor and a good functional liver reserve. However, a majority of HCC patients are often diagnosed at later stages when the tumors cannot be surgically removed [4]. Therefore, non-surgical treatments play an important role in the palliative treatment of HCC.

Hyperthermia (HT) is regarded as an effective technique to treat cancer by raising the temperature of the target tissue to approximately 40–45 °C [5]. HT has been practiced clinically for several decades, either as a monotherapy or in combination with other anti-cancer therapies [6, 7]. HT has been successfully used as one of the most potent sensitizers for chemotherapy and radiation treatment for various solid tumors [5–7]. The therapeutic effect of HT relies on destroying proteins and other components within the cells, changes in the tumor microenvironment, and stimulation of the immune response [8–10]. During local HT, precise temperature control throughout the entire target tissue is crucial to ensure a rapid, safe, and effective therapy [11]. However, several studies revealed dissipation of thermal energy from the blood flow of the target tissue that underwent HT when heating occurred near large vessels [11, 12]. This is a well-known phenomenon called the vascular heat-sink effect, which reduces the therapeutic effect of HT. One of the strategies to counter this involves blocking the blood flow at the region of interest (ROI) before HT to achieve a favorable therapeutic effect. For instance, transarterial embolization (TAE) assists in selectively reducing the blood perfusion and increasing the heated position. The rate of apoptosis in rabbits with renal VX2 carcinoma increased to 85.3 ± 4.7% by combining TAE with selective induction HT [13]. Therefore, the selective blocking of local blood flow is required, particularly for hypervascular tumors.

Microbubbles (MBs) are micron-sized spheres composed of a protein, lipid, or polymer shell, and a gas core [14, 15]. They pass through the lumina of capillaries and prevent extravasation from the vessels, which finally ensures their intravascular location [14, 15]. Therefore, these are commonly used as ultrasound contrast agents in ultrasound imaging for diagnostic purposes, which is referred to as contrast-enhanced ultrasound (CEUS) [16]. To date, several studies have been carried out on MBs as probes that reflect tissue perfusion by detecting the echogenicity of free MBs in the circulation using ultrasound [14–17]. In addition, MBs interact with therapeutic ultrasound (TUS) waves at high acoustic pressure with low acoustic intensities to promote inertial cavitation by providing a site for gas nucleation, for instance, microbubble-enhanced ultrasound (MEUS) [15, 18, 19]. Ultrasound-stimulated MBs oscillate constantly and violently until their implosion, collapse, or cracking of their encapsulated shells, which is accompanied by multiple behaviors of energy release, including extremely high pressure and temperature, shock waves, and high-speed microjets [15, 18, 19]. These events generate biological effects in vitro and in vivo, which include transient increases in nearby cell membrane permeability, severe mechanical injury in the small vessels, and the disruption of local flow, which significantly improve the potential for therapeutic applications [15, 20–22]*.* In addition, when MEUS is applied during a medical procedure, it is known as ultrasound cavitation treatment. Previous studies confirmed the MEUS-induced circulation cessation effect in the liver, which persisted for approximately 15–60 m [23], and this was considered to be enough time for ablation procedures that included percutaneous ethanol ablation (PEA) and radiofrequency ablation (RFA) [24, 25].

Based on MEUS-induced circulation blockage, the combination of HT and MEUS could result in a rapid and regular increment in therapeutic temperature in the liver tissue. Therefore, in this study, the potential, effectiveness, and safety of blocking liver perfusion that is mediated by MEUS to improve the therapeutic effect of HT in vivo were evaluated.

## Materials and methods

### Experimental animals and study design

In total, 40 healthy New Zealand rabbits that weighed 1.8–2.5 kg were purchased from the Guangdong Medical Laboratory Animal Center (Guangzhou, Guangdong, China). All the rabbits were reared at 24–26 °C under humidity of 45–55% for ≥ 7 days.

All the rabbits were randomly divided into four groups (*n *= 10 per group): TUS + MB + HT group, MB + HT group, normal saline (NS) + HT group, and MB + sham group. Before surgery, the rabbits were sedated with an intravenous injection of 3% sodium pentobarbital (Sigma Chemical, St. Louis, MO, USA) at 1.0 mL/kg. Tracheal intubation was performed to ensure artificial respiration and adequate oxygenation of the rabbits. After tracheal intubation, isoflurane was administrated to maintain anesthesia during the experiments. To reduce the incision pain, 0.1 mg/kg of buprenorphine (Temgesic; BD Pharmaceutical, Franklin Lakes, NJ, USA) was injected subcutaneously at the incision site, followed by the use of local anesthesia with 2 mg/kg of bupivacaine (Reyon Pharmaceutical, Seoul, South Korea). A heating pad was used to prevent a decrease in body temperature in the rabbits, which was monitored using a temperature probe that was placed in the rectum during the experiment. The animals were placed in a supine position, and the fur on the upper anterior abdomen was depilated. Then, a midline incision was made in the abdominal wall, followed by gently pulling out the left and middle lobes of the liver from the abdominal cavity and fixing them ex vivo in situ with saline-soaked gauze.

**Fig. 1:**
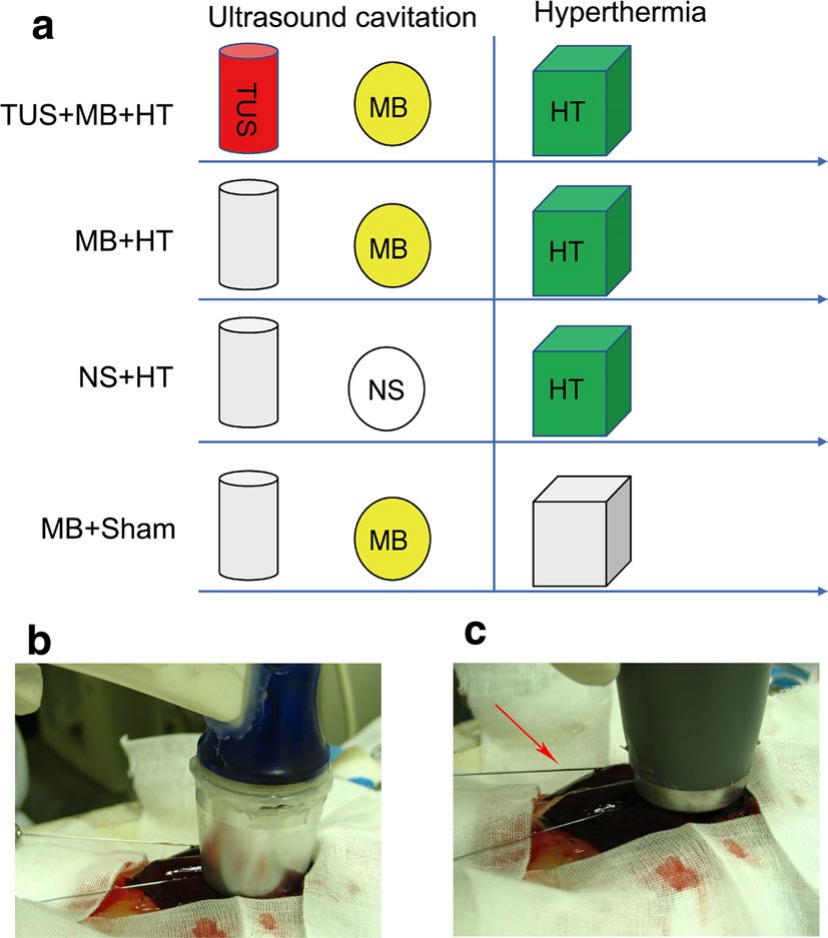
Experimental protocols and key procedures: **a** schematic representation of the study protocols; **b** ultrasound cavitation; and **c** HT procedure. Red arrow indicates thermocouple probe

During the procedures, MEUS was initially performed, followed by HT, as shown in Fig. 1a. In the TUS + MB + HT group, the targeted liver tissues were initially exposed to TUS for 5 m in the presence of an MB suspension at 0.1 mL/kg (dissolved in 5 mL NS), which was intravenously administrated, and then a standard HT procedure was performed in the same treated area. After the HT temperature reached 42 °C, the treatment was continued under the same HT conditions for 10 m. In the MB + HT group, MBs were intravenously administered, during which the targeted liver tissues were not exposed to TUS, and then the HT procedure was performed. In the NS + HT group, the same volume of NS was intravenously administered, during which the targeted liver tissues were not exposed to TUS, and then the HT procedure was carried out. In the MB + sham group, MBs were intravenously administered for 5 m, during which the targeted liver tissues were not exposed to TUS, and then a sham HT procedure was performed. The last three groups were treated with the same HT procedure as the TUS + MB + HT group, and the duration of the HT procedure depended on the corresponding individual rabbit in the TUS + MB + HT group. These groups were compared mainly to examine the effects of different treatments on the therapeutic effects of HT. CEUS was performed to observe the blood perfusion in the treated area at three timepoints: before treatment, immediately after ultrasound cavitation, and post-HT. During the HT procedure, the temperature of the treated and untreated areas was monitored by inserting two thermocouples into the liver. Blood samples from the rabbits were collected before the procedures and immediately after completion of the HT treatment to evaluate the levels of liver transaminases. Pathological changes in the liver tissues were observed after treatment by histological examination.

### MBs

Zhifuxian, which is a lipid-shelled MB contrast agent (Department of Ultrasound, Xinqiao Hospital, Chongqing, China) [26], was used in CEUS imaging and ultrasound cavitation treatment. It was prepared by lyophilization of a two-lipid suspension and then agitated with perfluoropropane gas using a high-speed mechanical amalgamator. The mean particle diameter of MBs was 2 μm. The concentration of MBs was approximately 2–9 × 10^9^/mL. Zhifuxian was shaken for 45 s and was intravenously administered in one injection at 0.01 mL/kg for CEUS imaging, and was continuously infused for 5 m at 0.1 mL/kg (dissolved in 5 mL NS) for MEUS.

### CEUS imaging and quantitative analysis

CEUS imaging of the liver was carried out at three timepoints: before treatment, immediately after MEUS, and post-HT. The Zhifuxian MBs were injected at 0.01 mL/kg as a bolus into the ear vein. However, the NS + HT group was continuously injected with NS, and equal amounts of MBs were used in the other groups before the HT procedure. CEUS imaging was performed post-HT in the NS + HT group. A commercial diagnostic ultrasound imaging system (GE Logiq 9; General Electric Company, New York, NY, USA) equipped with a 9L linear array transducer at a frequency of 5–9 MHz was utilized for CEUS imaging. Contrast imaging was performed in a coded enhanced harmonic manner with a mechanical index of 0.13. During this study, the image depth and the gain maintenance remained unchanged.

The CEUS images were recorded in DICOM format for quantitative analysis. The raw data were transformed into JPG images using Power Showcase software (Trillium Technology, MI, USA). CEUS images were transferred to the quantification software Adobe Photoshop CS3 (Adobe Systems Incorporated, San Jose, CA, USA), and re-sized with the sampling size of approximately 2 × 2 cm, equivalent to 6 × 6 mm in the original corresponding CECU images, and subsequently the grayscale value (GSV) of the images was analyzed. The GSVs were from 0 (black) to 255 (white). The sampling frame was located at a region of interest (ROI) that avoided large and medium-sized blood vessels. The average GSV of the ROI that reflected the liver blood perfusion was automatically calculated.

### Ultrasound cavitation

A standard procedure for ultrasound cavitation refers to TUS exposure combined with a continuous injection of MBs at the same time. An ultrasound cavitation apparatus with an ultrasound transducer (CZ960; Mianyang Sonic Electronic, Mianyang, China) was used. The transducer was weakly focused with a transducer diameter of 25 mm, and was operated at a frequency of 831 kHz, with an acoustic pressure (peak negative pressure) of 4.6 MPa, a pulse repetition frequency of 10 Hz, and a duty cycle of 0.5%. The acoustic intensity (I_STPA_) was approximately 0.89 W/cm^2^. The procedure was performed using an intermittent mode of 5 s on and 5 s off for 5 m. The transducer coupling with gel was gently placed over the liver (Fig. 1b). In the TUS + MB + HT group, the TUS exposure was combined with continuous intravenous injection of Zhifuxian MBs at a total dose of 0. 1 mL/kg (dissolved in 5 mL NS) for 5 m.

### HT

HT was performed using an ultrasound-mediated physiotherapy instrument (Metron Medical Australia Pty. Ltd., Carrum Downs, VIC, Australia). The shape of the transducer used for HT was weakly focused with a transducer diameter of 25 mm. It consisted of an ultrasound probe with a 1.1-MHz central frequency that transmitted pulses at a frequency of 100 Hz. The pulse width was 1.0 ms. The pulse interval was 10 ms. The acoustic intensity was approximately 1 W/cm^2^. As shown in Fig. 1c, the probe was placed vertically above the liver surface and remained motionless during the treatment.

### Temperature measurement

The temperature measurements were performed using two type K thermocouples with a probe diameter of 1.5 mm (Guangzhou Shenggao Measurement and Control Technology, Guangzhou, Guangdong, China). Before HT, one thermocouple was inserted into the treated liver and the other thermocouple was placed into the untreated liver at approximately 1 cm from the outer edge of the instrument probe to measure the baseline temperatures (Fig. 1c). The thermocouples recorded the real-time temperatures during HT.

### Blood sample collection

Blood samples from the femoral vein before the procedures and immediately after completion of HT were obtained from five rabbits per group to examine hepatic transaminases. During collection of the blood samples via the femoral vein, a soft, thin tube was placed in the femoral vein. Serum alanine aminotransferase (ALT) and aspartate aminotransferase (AST) levels were measured as indicators of liver damage. The differences between ALT and AST before and after the procedures were calculated.

### Pathological examination

All rabbits in each group were anesthetized by intravenous injection of sodium pentobarbital (120 mg/kg) and euthanized immediately after completion of the procedures. To assess the effects of different treatments on pathological changes of the liver, the liver tissue of three rabbits was fixed in formalin, embedded in paraffin, sectioned serially, and stained with hematoxylin and eosin (H&E). The liver cells, hepatic sinusoids, intrahepatic vessels, and the changes around them were observed under a high-power optical microscope (Axio Scope A1; Zeiss, Oberkochen, Germany).

### Statistical analysis

Continuous variables were summarized by presenting means and standard deviation. The data analysis from before and after treatment in each group for comparison was performed using a paired two-sample *t* test. Multiple comparisons between groups with equal variance (homogeneity of variance) were carried out using one-way analysis of variance (ANOVA), followed by Bonferroni correction. A nonparametric test was used for multiple comparisons of groups with unequal variances (nonhomogeneity of variance). The linear and quadratic regression equations for each group were obtained by considering HT time as an independent variable and the temperature of the treated areas as a dependent variable. Natural logarithms (ln) were calculated for ALT and AST to meet the normal distribution. All statistical analyses were performed using SPSS 19.0 (IBM, Armonk, NY, USA), and *p* values of < 0.05 were considered to be statistically significant. OriginPro (OriginLab Corporation, Northampton, MA, USA) was used for curve fitting. Graph Pad Prism 8 (GraphPad Software, San Diego, CA, USA) was used for statistical graphics.

## Results

### Quantitative analysis of CEUS images

**Fig. 2:**
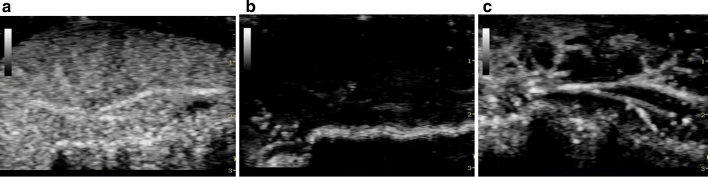
Contrast-enhanced ultrasound images of the liver in the TUS + MB + HT group. The treated liver lobe showed homogeneous enhancement before treatment (**a**); Liver perfusion was temporarily blocked after MEUS (**b**), and partially recovered after HT (**c**)

As given in Table 1 and Fig. 2, there was no significant difference in the GSV between the groups with pretreatment. Of note, in the TUS + MB + HT group, the GSV decreased significantly after the ultrasound cavitation procedure (0.42 ± 0.29 versus 119.52 ± 4.14; *p* < 0.01) or HT treatment (2.18 ± 0.68 versus 119.52 ± 4.14; *p* < 0.01) compared with that before treatment. However, no significant difference was observed in the GSV before and after treatment in the MB + HT or MB + sham group.

**Table 1:** Comparison of GSV between groups

Groups	Pretreatment	After ultrasound cavitation	Post-HT
TUS + MB + HT	119.52 ± 4.14	0.42 ± 0.29**	2.18 ± 0.68**
MB + HT	116.42 ± 6.18	116.53 ± 5.88	112.98 ± 7.73
NS + HT	–	–	118.30 ± 3.87
MB + sham	117.09 ± 5.45	116.90 ± 4.99	117.03 ± 4.81

**p < 0.01 compared with the pretreatment value in the TUS + MB + HT group

### Temperature changes during HT

**Fig. 3:**
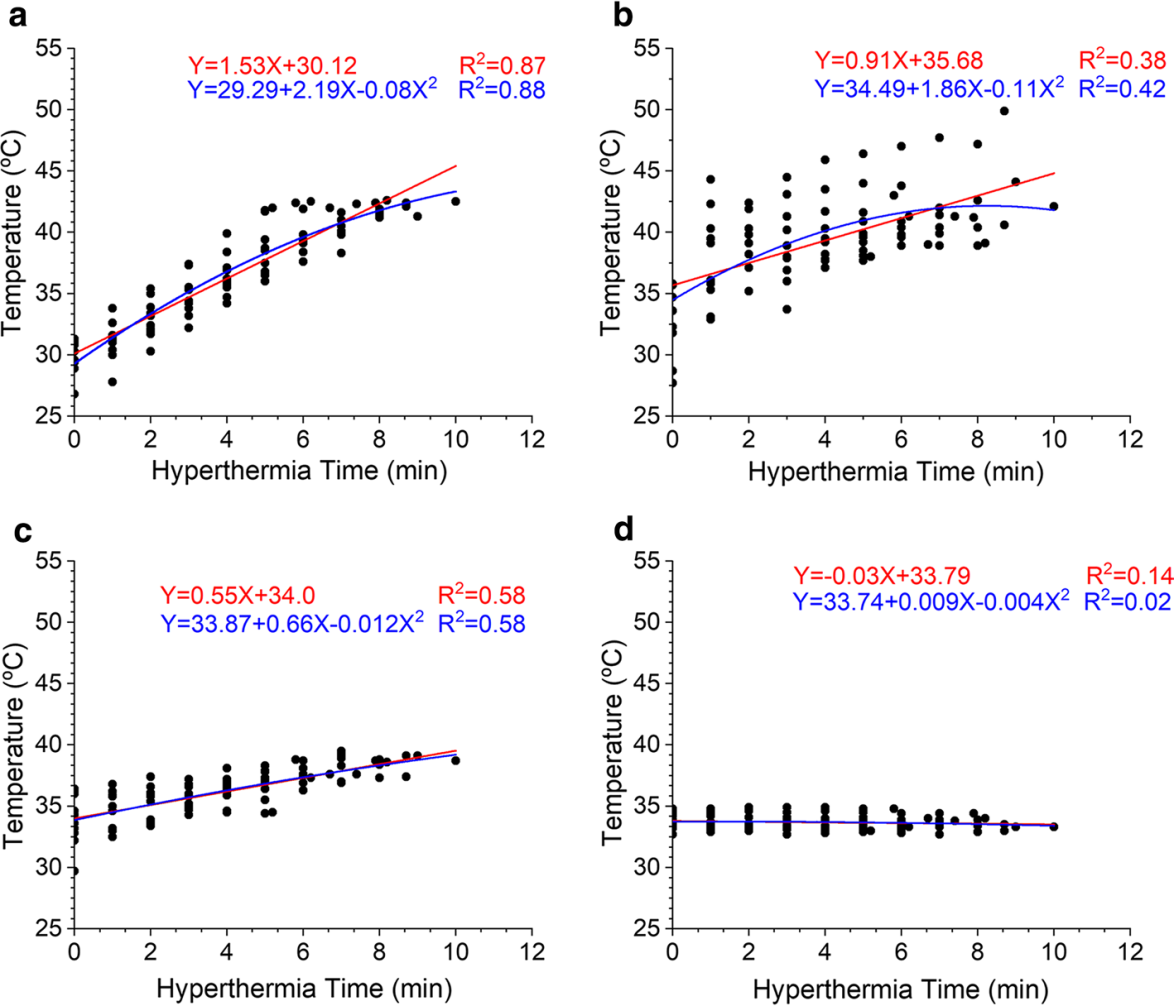
Linear and nonlinear curve fitting of the relationship between temperature of the targeted areas and HT time: **a** TUS + MB + HT group; **b** MB + HT group; **c** NS + HT group; and **d** MB + sham group

From baseline temperature to 42 °C, the linear and nonlinear regression equations were obtained and used to assess the relationship between HT time and temperature in different treatment groups (Fig. 3). In addition, in the linear fitting models or nonlinear fitting models of the four experimental groups, the regression equation for the TUS + MB + HT group indicated the strongest relationship between HT time and temperature (*R*^2^ = 0.87 for the linear regression model; *R*^2^ = 0.88 for the nonlinear regression model). These results indicated significantly higher temperatures in the treated areas in the TUS + MB + HT and MB + HT groups versus the other groups, and the TUS + MB + HT group exhibited more stable and regular fitting curves compared with the MB + HT group.

**Fig. 4:**
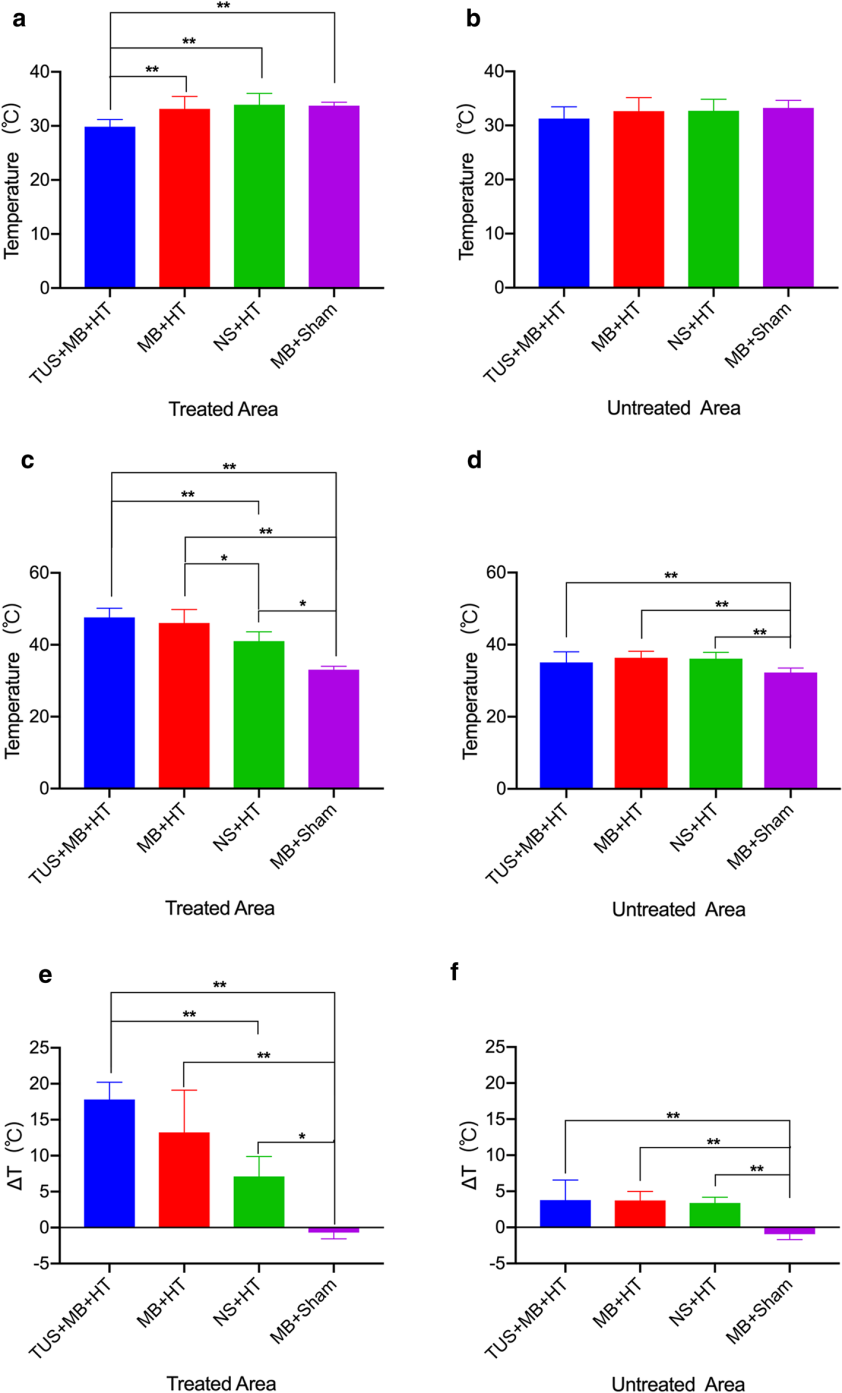
Comparison of temperatures in the treated areas and untreated areas of different experimental groups. The temperatures of the treated areas and untreated areas immediately after starting HT as baseline temperatures and on completion of HT as post-HT temperatures were measured using thermocouples. Baseline temperatures in the treated areas (**a**) and the untreated areas (**b**) in the TUS + MB + HT group, MB + HT group, NS + HT group, and MB + sham group; Post-HT temperatures in the treated areas (**c**) and the untreated areas (**d**) in the TUS + MB + HT group, MB + HT group, NS + HT group, and MB + sham group; Changes in temperatures in the treated areas (**e**) and the untreated areas (**f**) from baseline to post-HT in the TUS + MB + HT group, MB + HT group, NS + HT group, and MB + sham group. *p < 0.05 indicates a significant difference between groups; **p < 0.01 denotes a significant difference between groups

The temperatures of the treated areas and untreated areas at baseline and post-HT in the different treatment groups are shown in Figs. 4a–c, and d. On completion of HT, the temperatures in the treated areas were significantly higher in the TUS + MB + HT group compared with the NS + HT or MB + sham as the control group. The temperatures of the treated areas significantly increased in the TUS + MB + HT and MB + HT groups; however, there was no statistical difference in the post-HT temperatures between the TUS + MB + HT and MB + HT groups. In addition, a comparison of the temperature difference revealed a significantly larger temperature increase in the TUS + MB + HT group compared with the other three groups with a negative value observed in the MB + sham group (Fig. 4e).

Similarly, the temperature difference in the untreated areas in the MB + sham group showed a negative value. Compared with the MB + sham group, the TUS + MB + HT, MB + HT, and NS + HT groups induced significant increases in the post-HT temperatures in the untreated areas (Fig. 4f). However, the post-HT temperature difference in the untreated areas between these three groups (TUS + MB + HT, MB + HT, and NS + HT groups) showed no statistical significance.

In addition, all the rabbits in the MB + sham group failed to reach 42 °C after HT treatment, and 40% (4/10) of rabbits in the NS + HT group failed to reach the specified temperature of 42 °C. In addition, comparison of the time required to reach 42 °C between the TUS + MB + HT and MB + HT groups was not significant.

### Changes in levels of liver transaminases

**Fig. 5:**
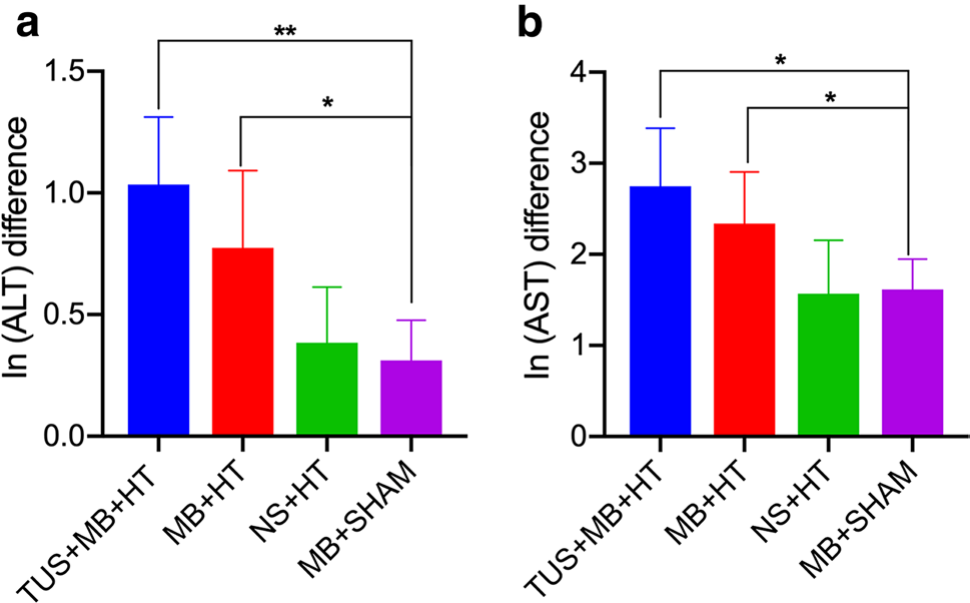
ln (ALT) and ln (AST) differences in each group: **a** comparison of ln (ALT) difference and **b** comparison of ln (AST) difference. *p < 0.05 indicates a significant difference between groups; **p < 0.01 denotes a significant difference between groups

The changes in serum levels of liver transaminases (ALT, AST) are shown in Fig. 5. There were significant differences in the changes in ALT (Fig. 5a) and AST (Fig. 5b) between the four different treatments (*p* < 0.05). Of interest, ln (ALT) and ln (AST) differences were significantly higher in the TUS + MB + HT group than those in the NS + HT group and MB + sham group. However, there were no significant ln (ALT) and ln (AST) differences between the TUS + MB + HT and MB + HT groups.

### Pathological examinations

**Fig. 6:**
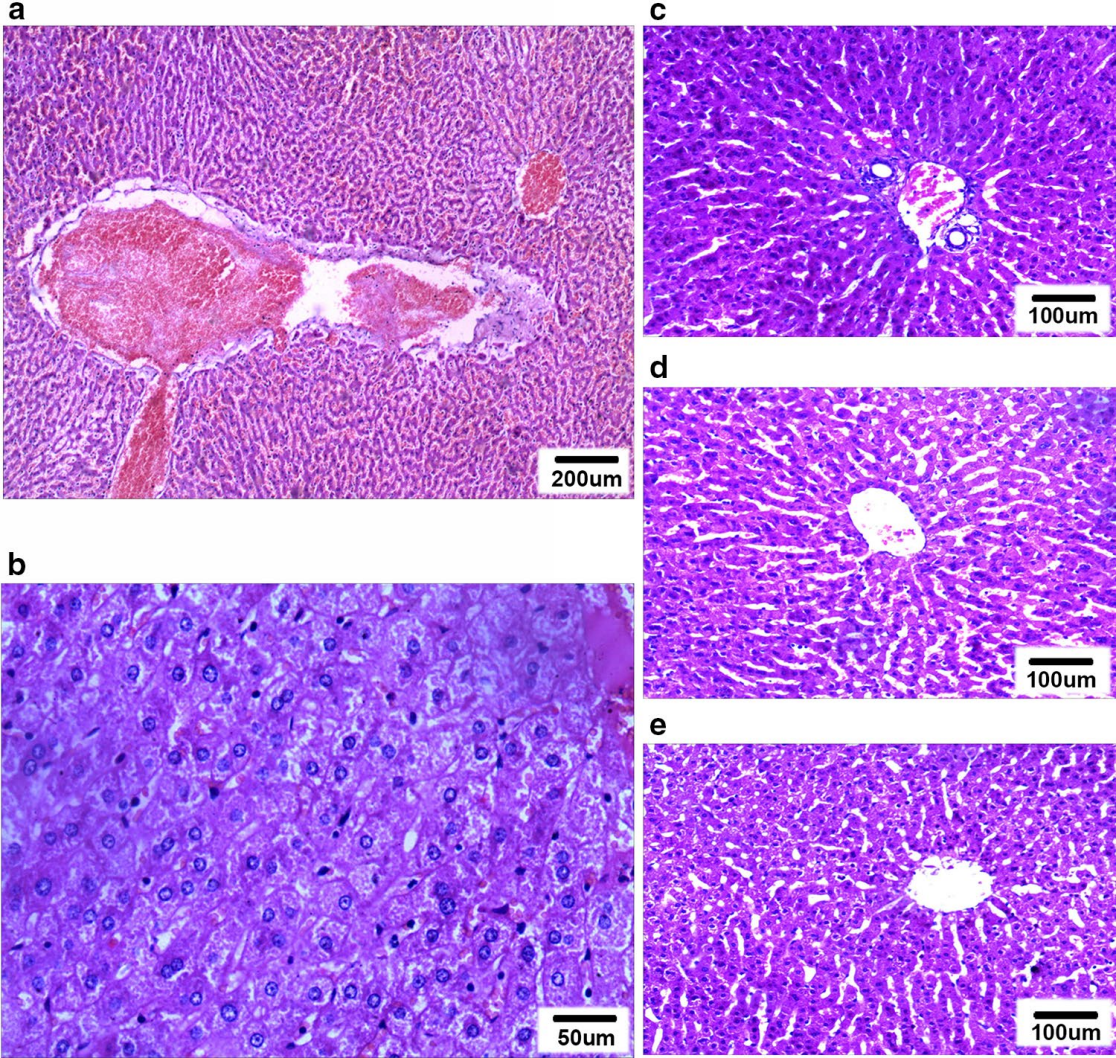
Pathological images of treated liver tissues with H&E staining: **a** extensive sleeve-like hematoma at the portal areas in the TUS + MB + HT group; **b** swelling hepatocytes compressed and shrank the intervening sinusoids in the TUS + MB + HT group; **c** some erythrocytes accumulated in the portal areas in the MB + HT group; **d** some erythrocytes accumulated in the portal areas in the NS + HT group; and **e** structure of the liver cells and visible sinusoids in the MB + sham group appeared normal

For the TUS + MB + HT group, two kinds of pathological changes were observed. First, a diffuse hemorrhage and focal thrombus were observed in the portal areas. When the hemorrhage occurred in the connective tissue layer of the portal canals, it often formed a circular and sleeve-like hematoma that surrounded the portal veins, although the structure of the portal wall appeared intact (Fig. 6a). Second, the hepatocytes were swollen and squeezed the sinuses around them (Fig. 6b). Some erythrocytes had accumulated in the portal areas in the MB + HT group (Fig. 6c) and NS + HT group (Fig. 6d), with a higher number of erythrocytes in the MB + HT group than in the NS + HT group. For the MB + sham group, normal liver cells that were arranged in plates with visible sinusoids in between were observed (Fig. 6e).

## Discussion

This study found that the temperature of the targeted areas in the liver increased more rapidly and steadily to 42ºC during treatment with MEUS, which led to swelling of the liver cells, compression of the sinusoids, and hemorrhage and thrombosis of the portal area; however, no severe liver damage was observed from the treatment. This could assist when determining the enhancing effects of MEUS on HT of the rabbit liver in vivo.

As mentioned in previous studies, MEUS using microbubble-enhanced acoustic cavitation could temporarily reduce or shut down the circulation of the liver, spleen, and solid tumors [23, 24, 27, 28]. The underlying mechanism is closely related to the mechanical effects of MEUS, i.e., high pressure, shock waves, and microjets, which results in the increased transient permeability of the cell membrane (sonoporation) and the injury of vascular endothelial cells [20, 22]. Then, microvascular disruption, tissue edema, hematoma formation, and thrombosis occur in the target tissues [21, 23], which reduces or blocks blood perfusion in the relevant tissues [21, 23]. Lower blood circulation is accompanied by lower heat loss. Therefore, these anti-vascular effects could provide a potential opportunity to overcome the heat-sink effect.

In the TUS + MB + HT group, the blood perfusion to the treated areas decreased after exposure to MEUS for 5 m before HT, which was supported by a significant GSV decline (Table 1, Fig. 2b), and pathological changes. Swollen and cloudy hepatocytes compressed the sinusoids and perisinusoidal space in the treated area, and massive hemorrhaging and focal thrombosis in the portal area raised the pressure in the portal channel. These pathological results agreed with the findings in previous studies [23, 29]. In addition, TUS + MB + HT combination treatment involved an optimal fitting curve of the temperature differences and the largest temperature change. These findings agreed with those reported previously [23, 29]. In normal liver tissues, MEUS interrupts the blood perfusion to the targeted areas, which results in the deposition of more heat during ablation, and enlarges the PEA volume up to 10 times and the RFA volume up to 2.8 times [24, 25]. In addition, it was confirmed that the combination of MEUS and PEA significantly improved the necrosis rate of rat Walker 256 tumor cells from 80.0% to 97.5% [28]. Based on this previous study, MEUS could facilitate HT through MEUS-mediated disruption of blood perfusion to the targeted areas of the liver and could overcome the heat-sink effect. The reduction in the heat-sink effect could enhance the deposition of heat and help to maintain a rapid and regular increase in the temperature within the targeted areas of the liver, and therefore, the effectiveness of HT could be improved.

In this study, the MB + HT group showed a temperature increase, which was not stably enhanced compared with the TUS + MB + HT group. The exact mechanisms remain unclear and require further investigation. Based on previous studies of MB contrast agents, intravenously injected MBs before HT changed the acoustic environment of the target tissue and increased the acoustic impedance difference between the target tissue and surrounding tissue. During HT, the residual MBs that were exposed to the ultrasonic field increased the deposition of ultrasonic energy and enhanced the thermal effect of ultrasound. This caused an increase in temperature in the target tissue. However, blood flow in the target tissue led to an uneven local distribution of microbubbles, which resulted in fluctuations in temperature. Unlike the MB + HT group, MBs were initially exposed to low-intensity ultrasound in the TUS + MB + HT group, which led to the desirable non-thermal cavitation effects and blocking the blood perfusion to the target areas of the liver, as reported previously [23–25]. On completion of TUS + MB and achieving a reduced heat-sink effect, the targeted liver tissues then underwent HT, which led to the thermal effects, gradual increase in temperatures, and the maintenance of a stable high temperature. The findings from this study support the previous possibilities. As shown in Figs. 3 and 4, the TUS + MB + HT group exhibited a more stable and regular increase in temperatures in the fitting curves compared with the MB + HT group.

In addition, the effect of the treatment on the change in temperature in the untreated areas remains a concern. The temperature difference in the untreated area showed a negative value in the MB + sham group, which suggested that the decrease in temperature could be associated with a sham HT. In contrast, the remaining three groups demonstrated positive temperature differences in the untreated areas but showed no statistically significant differences between these groups. These results suggested that the combination of MEUS did not increase the differences in the temperature in the untreated tissues, which demonstrated the safe use of MEUS in combination.

In this study, serum ALT and AST levels, which are indicators of liver damage, were monitored. After HT treatment, there were apparent increases in serum ALT and AST levels in the TUS + MB + HT and MB + HT groups compared with those in the NS + HT and MB + sham groups as controls, and these elevated levels might signify liver damage from the treatment. Although hepatocellular injury in the TUS + MB + HT group might have led to elevated levels of these transaminases, the findings from this study and those in the literature supported the notion that the liver injury that was caused by the regional and temporal liver blood perfusion was acute and could be restored [23, 24]. These results combined with those in previous studies suggested the safety of the combination of MEUS and HT to treat the liver [23, 24]. In addition, it was reported that the peak of ALT and AST occurred 24–48 h after treatment in the MEUS + RFA and RFA groups, and that their levels then decreased and returned to the normal ranges 8 days following treatment [24]. This study only monitored the changes in ALT and ASL levels for liver injury immediately after treatment; therefore, further studies are needed to determine if and when the hepatic transaminase levels return to normal levels.

In the present study, the use of MEUS was preliminarily explored to enhance the effects of HT. Therefore, the MEUS technique was successfully applied with clinical utility, and future studies should investigate this technique to obtain the maximum therapeutic effect with minimal side effects. Future research should first focus on the immediate effects of MEUS on the liver in combination. Although the immediate effects helped our understanding of the effects of MEUS-mediated changes in the liver vasculature and perfusion, further studies into the bioeffects should be conducted to assess at prolonged timepoints whether the treatment contributes to the injury responses of liver tissue and the surrounding organs for safety. Second, the MEUS technique should be tested in large animals, or various orthotropic tumors, or both, which could serve as preclinical studies. Finally, the acoustic parameters and pulse sequences of MEUS delivery could be optimized to improve the therapeutic response and to maintain stability.

## Conclusion

The effectiveness of HT could be enhanced in combination with MEUS in normal liver tissues. The mechanism of this synergistic effect demonstrated a close relationship with blood perfusion that is blocked by MEUS.
